# Esophageal Perforation During Dilation in a Patient With Hypopharyngeal Metastatic Squamous Cell Carcinoma

**DOI:** 10.7759/cureus.115311

**Published:** 2026-08-27

**Authors:** David Ai, Mauna I Rao, Ashley C Wetzig

**Affiliations:** 1 Department of Anesthesiology, Baylor College of Medicine, Houston, USA

**Keywords:** anesthesia, esophageal stricture, hypopharyngeal cancer, iatrogenic esophageal perforation (iep), pneumoperitoneum, pneumothorax, secondary pneumomediastinum

## Abstract

We report the case of a 75-year-old female with a history of stage IVa hypopharyngeal squamous cell carcinoma who presented with dysphagia due to cervical esophageal stricture after prior laryngopharyngectomy and cervical esophagectomy. During rigid pharyngeal dilation, an acute and uncontained cervical esophageal perforation involving the superior mediastinum occurred, leading to pneumomediastinum. This was immediately complicated by her surgically altered anatomy creating bilateral pneumothoraces, tension pneumoperitoneum, and subsequent hemodynamic instability. Prompt recognition and emergent intervention, including needle decompression, chest tube placement, exploratory laparotomy, and intensive postoperative care, stabilized the patient’s condition. This case highlights the challenges in managing high-risk patients with complex airway and esophageal conditions, underscoring the importance of multidisciplinary coordination and rapid response in preventing fatal outcomes.

## Introduction

Esophageal perforation is a rare but serious complication that can arise during therapeutic interventions such as esophageal dilation, particularly in patients with a history of malignancy. A perforation allows air and gastrointestinal contents to escape the esophagus into surrounding spaces, including the mediastinum, pleura, or peritoneum. This can rapidly lead to severe infection, hemodynamic collapse, and cardiac arrest. Malignant esophageal strictures, seen in patients treated for head and neck or esophageal cancer, are especially vulnerable to injury because prior surgery, radiation, and chemotherapy weaken normal tissue integrity [1,2]. Other commonly observed severe complications of esophageal dilations include hemorrhage, bacteremia, and sepsis [3].

Population data estimate the incidence of esophageal perforation to be 3.1/1,000,000 person-years and an overall mortality rate of 13.3%, influenced by the cause of perforation and time to diagnosis [4]. Roughly half of perforations are iatrogenic and spontaneous occurrences [5,6]. Perforations are also categorized by anatomic location, as the esophagus spans the cervical, thoracic, and abdominal compartments. Most occur in the thoracic cavity (72.6%), followed by cervical (15.2%) and abdominal (12.5%) [5]. This distinction is clinically important: thoracic perforations can rapidly contaminate the mediastinum and pleural space due to negative intrathoracic pressure, whereas cervical perforations more commonly present with subcutaneous emphysema [7]. Delayed recognition markedly worsens outcomes, with mortality doubling when diagnosis is delayed beyond 24 hours [2,5].

The incidence of perforation during routine esophageal dilation procedures is overall low (0.09% - 0.7%), but as was present in the following described case, invasive malignancy, prior surgery, and radiation therapy significantly increase that potential risk [8]. The American Society for Gastrointestinal Endoscopy highlights that the presence of head and neck cancer and dilation of malignant strictures significantly increases the risk of perforation [8]. Diagnosis can be challenging, as early symptoms may be nonspecific and initial imaging unrevealing, necessitating a high index of suspicion and repeat or extended evaluation [9].

Treatment of esophageal perforation depends on perforation location, containment, and patient factors, ranging from conservative therapy to endoscopic or surgical intervention [10,11]. Perforations can also be localized by contrast studies. However, there is no consensus for the best treatment [12]. Overall goals of management include aggressive fluid resuscitation, broad-spectrum antibiotics, nutritional support, nil per os (NPO), and eventual restoration of gastrointestinal continuity [10,12]. In recent years, nonoperative measures have become more common for patients with well-defined tears and minimal extraesophageal involvement [9,10]. The American Gastroenterological Association notes that minimally invasive techniques, including stenting and clipping, are increasingly used for contained perforations, while it remains essential to consider open surgical intervention for cases of uncontained air leak, extensive contamination, sepsis, delayed diagnosis, or respiratory complications such as pneumothorax and respiratory failure [10,13]. Preoperatively, perforation localization, broad-spectrum antibiotics, and fluid resuscitation are still crucial. Invasive interventions include surgical repair, endoscopic stenting, or subtotal esophagectomy in the most severe cases [10,12].

This case emphasizes, from the anesthesiologist's perspective, the challenges of recognizing acute cervical esophageal perforation involving the superior mediastinum in a high-risk patient with prior laryngectomy and tracheoesophageal puncture (TEP). It underscores the unique use of the patient’s TEP in initial detection of esophageal perforation, and it highlights the importance of early recognition, interdisciplinary communication, and rapid intervention when caring for intraoperative patients with complex oncologic and surgical histories. This article adheres to the Case Report (CARE) guideline. Written Health Insurance Portability and Accountability Act (HIPAA) authorization was obtained from the patient.

## Case presentation

A 75-year-old female with a history of stage IVa hypopharyngeal squamous cell carcinoma treated with chemotherapy, radiation, total laryngopharyngectomy, and cervical esophagectomy presented with worsening dysphagia. Of note, she also had a TEP with a voice prosthesis for phonation. She had had multiple esophagogastroduodenoscopies (EGDs) and serial dilations for cervical esophageal stenosis. The most recent EGD by the gastroenterology team was unsuccessful in passing a guidewire through the stricture, so the patient was referred to the otolaryngology team for further exploration and dilation in the operating room.

During her planned direct pharyngoscopy with rigid pharyngeal dilation, a 6.0 wire-reinforced endotracheal tube was placed through the laryngeal stoma, with placement confirmed by bronchoscopy, which also found no active esophageal bleeding. Then, dilators ranging from 10 Fr to 20 Fr were passed without resistance. While inserting the 22 Fr dilator, resistance was encountered, and dilation was immediately discontinued. A pediatric bronchoscope was introduced through the endotracheal tube to visualize the esophagus. Auto‑insufflation was used to improve visualization; however, the bronchoscope encountered resistance at the TEP site and could not be advanced. Shortly thereafter, ventilation became difficult, and oxygen saturations rapidly dropped into the 70s. The chest and abdomen were noted to be firm, and breath sounds were faint to absent bilaterally. An anesthesia stat was called overhead as end-tidal CO₂ (ETCO₂) decreased to zero and mean arterial pressure (MAP) decreased to the 50s. Needle decompression of the superior chest was performed without significant air return. Chest wall crepitus was discovered, and the patient became progressively hypotensive and tachycardic. An arterial line was promptly placed, and vasopressors were escalated, maintaining MAP up to the 110s.

**Figure 1:**
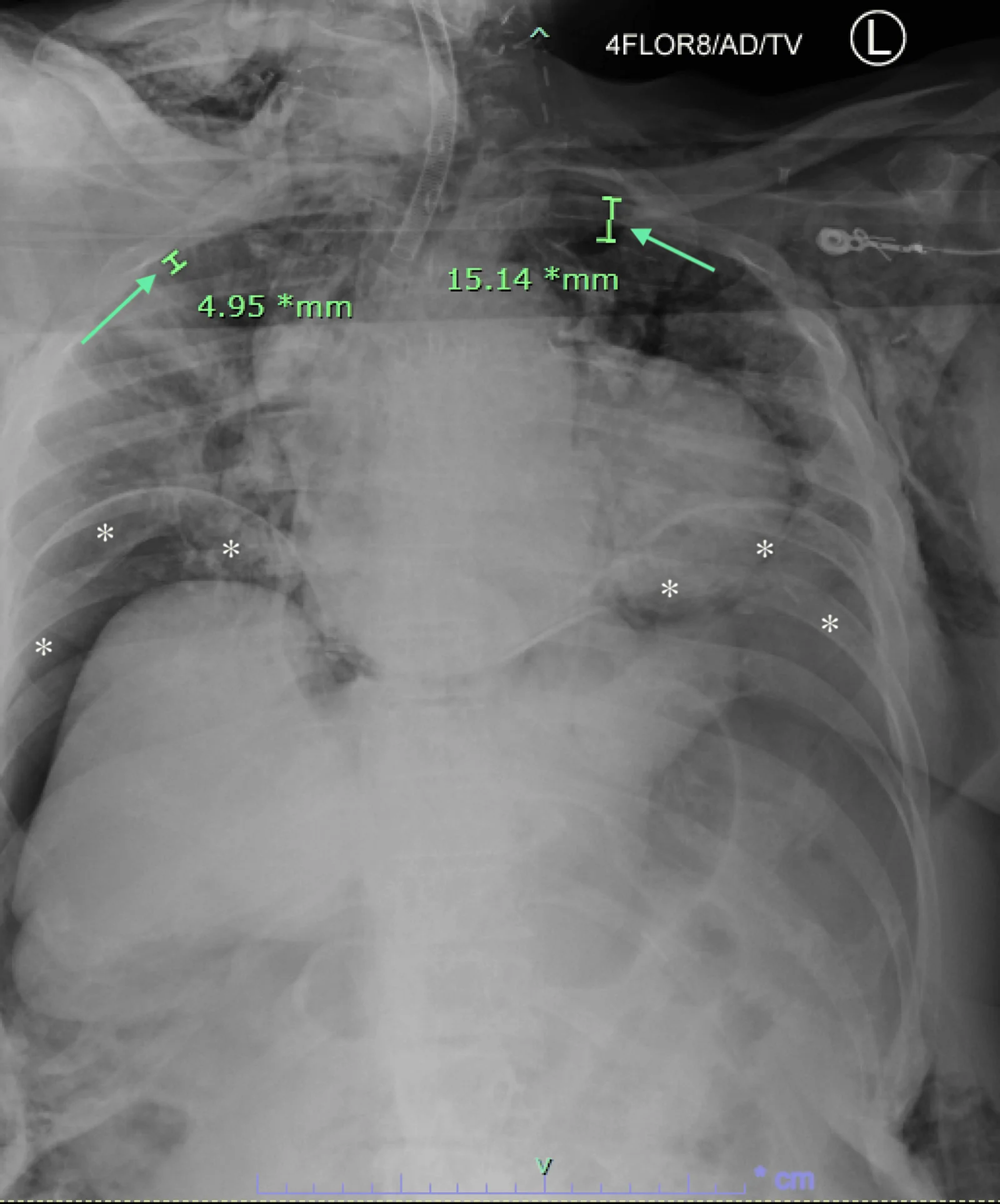
Chest radiograph Showing small bilateral pneumothoraces (green arrows) and extensive pneumoperitoneum (white stars).

A subsequent stat chest radiograph demonstrated bilateral tension pneumothoraces, pneumomediastinum, and extensive pneumoperitoneum (Figure 1). General surgery and cardiothoracic surgery were consulted intraoperatively, as esophageal perforation was highly suspected. Central venous access was obtained via a right femoral cutdown due to difficulty accessing the neck veins. Bilateral chest tubes were placed to release air from the pneumothorax, followed by an emergent exploratory laparotomy, which resulted in the release of air from the pneumoperitoneum. The patient’s ventilation and hemodynamics improved significantly, with ETCO_2_ stable in the 30s, MAP up to the 110s, and heart rate between 90 and 110 beats per minute.

**Figure 2:**
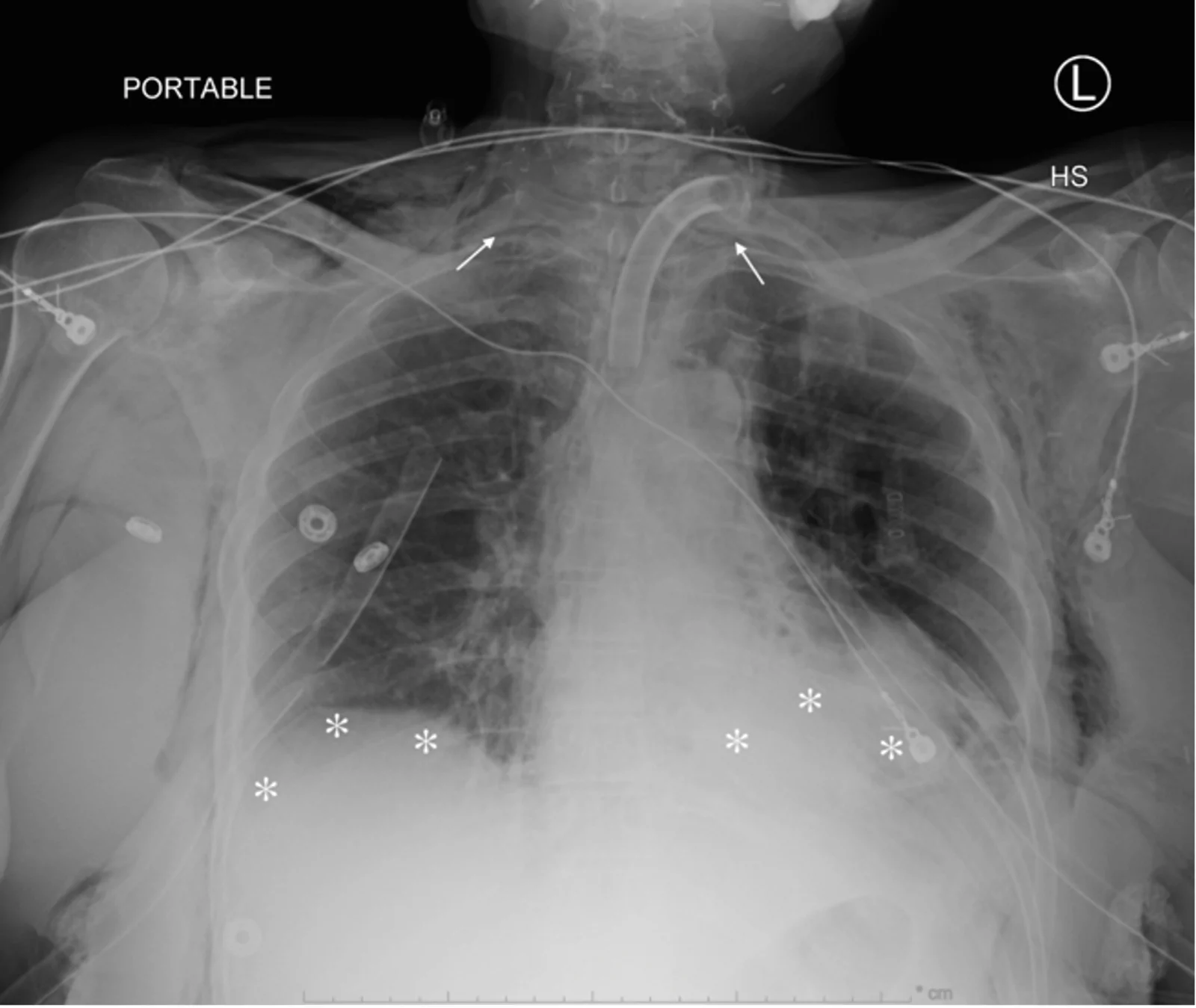
Follow-up chest radiograph Reveals resolved bilateral pneumothoraces with lung markings present in both superior lobes (white arrows) and resolved pneumoperitoneum, as the top of the diaphragm (white stars) is not surrounded by free air. Of note, the heart is no longer pushed upwards due to the pneumoperitoneum (Figure 1). HS: hora somni

A chest radiograph obtained after intervention demonstrated resolution of the majority of air in the mediastinum, pleura, and peritoneum (Figure 2). The procedure was aborted, and the patient was started on broad-spectrum antimicrobial therapy. 

**Figure 3:**
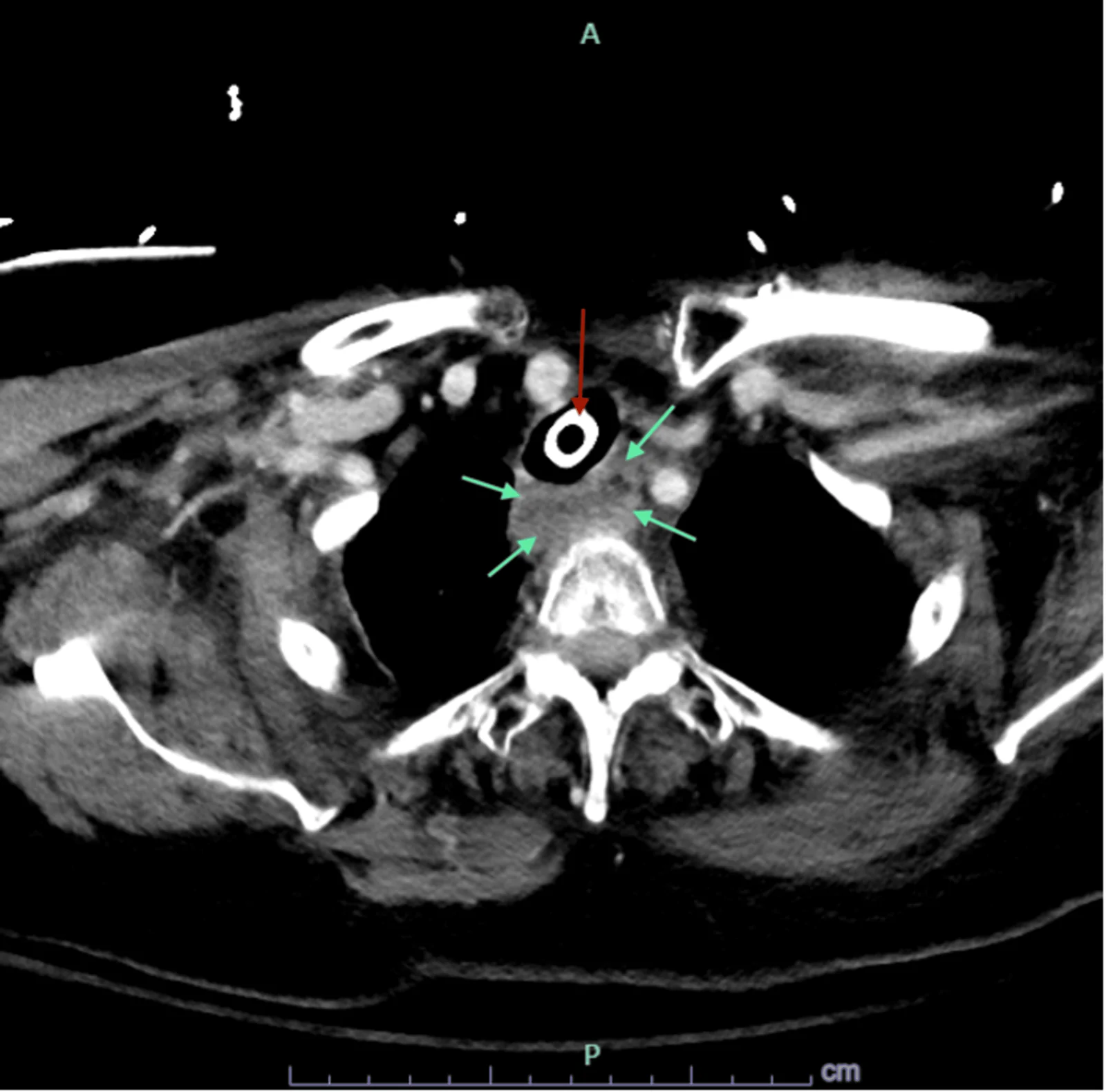
CT scan with contrast Circumferential esophageal edema post-esophageal perforation (green arrows). The patient’s tracheostomy tube (red arrow) can be seen in the trachea. A: anterior; P: posterior

Postoperatively, the patient required ICU care, including broad-spectrum antibiotics, ventilator support, and vasopressors. A CT scan with contrast was performed one day after the perforation and ultimately revealed circumferential edema involving the proximal esophagus consistent with the recent perforation (Figure 3). The patient underwent additional procedures, including abdominal wound closure and placement of a gastrostomy-jejunostomy (GJ) tube. She was discharged to a long-term acute care facility and subsequently transitioned to home care, remaining NPO with enteral feeding. Informed written consent was obtained from the patient for the open-access publication of this case report.

## Discussion

This case is notable for the rapid intraoperative recognition of an acute cervical esophageal perforation in a patient with complex post-head and neck cancer anatomy, including prior laryngectomy and TEP. The tissue, altered by previous radiation and surgery, likely predisposed the esophagus to perforation during dilation, resulting in rapid release of air into the mediastinum, pleural spaces, and peritoneum. A distinguishing element of this case is the early clue provided by reversal of airflow through the TEP. Because the TEP functions as a one-way valve allowing air from the lungs into the esophagus for phonation, the observed backward airflow strongly suggested an abnormal presence of air in the esophagus. In this setting, pneumoperitoneum and pneumomediastinum likely overcame the valve mechanism, serving as an early and unusual indicator of esophageal rupture. The patient’s acute desaturation and escalating ventilatory pressures further supported this diagnosis, as the development of chest crepitus and diminished breath sounds was consistent with rapidly evolving bilateral pneumothoraces in the setting of esophageal perforation. This sequence highlights an important mechanistic consideration: increased ventilation can exacerbate air dissection from an uncontained esophageal injury, accelerating pleural compromise and hemodynamic instability.

Airway management in patients with prior laryngectomy and TEPs requires special considerations, as standard intubation techniques are not feasible. It is crucial to remember that these patients cannot be oxygenated, ventilated, or intubated from above the stoma. In this case, the patient’s endotracheal tube was sutured to the stoma and remained at the same depth since confirmation of placement with bronchoscopy at the start of the case, decreasing the possibility of tube migration.

The initial attempt to visualize the esophageal perforation via bronchoscopy is supported by the current literature, as direct visualization of structures beyond the esophagus, such as the lungs, is diagnostic of esophageal perforation [14]. Although CT scans have high sensitivity for locating the perforation [14], the intraoperative nature and acute difficulties managing the patient’s ventilations and hemodynamics were not conducive to obtaining a CT scan. Plain chest radiographs may be useful in locating the perforation if the patient can swallow water-soluble contrast, which was also not possible in this acute setting. Without contrast, chest radiographs can show signs suggesting esophageal injury, including pneumothorax, lung collapse, pneumediastinum, and subcutaneous emphysema [14], many of which were present in Figure 1.

Bilateral needle decompression for pneumothorax in this patient may have prevented cardiopulmonary collapse in this patient while awaiting chest tube placement by the trauma surgeons. Vascular access also posed significant challenges due to prior surgeries and radiation treatments to the neck area. Avoidance of the internal jugular veins and failed bilateral subclavian attempts, likely related to radiation-induced stenosis, necessitated femoral vein cutdown. This illustrates the importance of anticipating limited access options in patients with advanced head and neck cancer and preparing early for alternative strategies.

A key lesson from this case is the importance of maintaining a high index of suspicion for esophageal perforation when sudden ventilatory or hemodynamic changes occur during dilation in high-risk patients. This is especially important since the low incidence of esophageal perforations makes it difficult for the clinician to acquire extensive clinical experience in managing such emergencies. The challenges of obtaining confirmatory findings of esophageal perforation can also make detection difficult.

The interdisciplinary collaboration between the anesthesia, general surgery, cardiothoracic surgery, otolaryngology, and radiology teams facilitated a rapid and well-coordinated response. The general surgeons swiftly performed chest tube placement following needle decompression, mitigating the risk of tension pneumothorax and cardiovascular collapse. The coordinated use of bronchoscopy and chest radiography provided timely confirmation of tube position and extent of air dissection, guiding immediate management.

This report is limited by its single-case nature and the absence of direct intraoperative visualization of the perforation at the time of initial decompensation. Nonetheless, it contributes to the literature by highlighting an uncommon but clinically meaningful warning sign, reversal of airflow through a TEP, and reinforces the need for vigilance, rapid escalation, and team-based response when caring for patients with complex oncologic airway and esophageal anatomy.

## Conclusions

A prompt multidisciplinary approach in recognizing and managing high-risk patients with complex surgical and oncologic histories was critical. Each patient is unique, and this case report demonstrates the unique utilization of the direction of TEP airflow for detecting increased thoracic pressure and developing suspicion for esophageal perforation. The use of imaging, though standard practice, corroborated the TEP findings and physical exam. Flexibility in obtaining vascular access permitted successful hemodynamic support in this patient with extensive surgical and radiation history. Ensuring effective communication and collaboration across specialties remains vital to optimizing patient outcomes in such life-threatening scenarios.
